# Metastatic breast cancer in Kenya: survival, prognosis and management at a tertiary referral centre

**DOI:** 10.3332/ecancer.2023.1566

**Published:** 2023-06-27

**Authors:** Mwongeli Matheka, Miriam Mutebi, Shahin Sayed, Jasmit Shah, Asim Jamal Shaikh

**Affiliations:** 1 Aga Khan University Hospital Nairobi, PO Box 30270 - 00100, Nairobi, Kenya; 2 Department of Medicine, Aga Khan University, PO Box 30270 - 00100, Nairobi, Kenya; 3 Brain and Mind Institute, Aga Khan University, PO Box 30270 - 00100, Nairobi, Kenya; 4 Sultan Qaboos Comprehensive Cancer Care and Research Centre, Muscat, Oman

**Keywords:** breast neoplasms, metastasis, survival rate

## Abstract

There has been an increase in breast cancer in Africa with up to 77% of patients diagnosed with advanced disease. However, there is little data on survival outcomes and prognostic factors affecting survival in patients with metastatic breast cancer (MBC) in Africa. The study objective was to establish the survival of patients with MBC at a single tertiary health facility, the clinical and pathological characteristics affecting survival and describe the treatment modalities used. This was a retrospective descriptive study conducted at Aga Khan University Hospital, Nairobi of patients diagnosed with MBC between 2009 and 2017. Survival data was collected on metastatic free survival, survival time between diagnosis of first metastasis and death and overall survival. Data on patient’s age, menopausal status and stage at diagnosis, tumour grade, receptor status, site of metastasis and treatment given was also collected. The Kaplan-Meier Estimator was used to estimate survival. Prognostic factors for survival outcomes were analysed using univariate analysis. Standard descriptive statistics were used to describe patient characteristics.

A total of 131 patients were included in the study. The median survival was 22 months. The 3 and 5-year survivals were 31.3% and 10.7%, respectively. On univariate analysis, the Luminal A molecular subtype was a significant positive prognostic factor hazard ratios (HR 0.652 95% confidence interval (CI) 0.473–0.899) while metastasis to the liver or brain were significant negative prognostic factors (HR 0.615 95% CI 0.413–0.915 and HR 0.566 95% CI 0.330–0.973, respectively). A large proportion (87.0%) received some treatment for metastatic disease. Our study concluded that survival rates for patients diagnosed with MBC were lower compared to studies from Western countries but higher than in studies from Sub-Saharan Africa. Luminal A molecular subtype was found to be a positive prognostic factor and metastasis to the liver or brain were found to be negative prognostic factors. Improved access to adequate treatment for MBC is required in the region.

## Introduction

Although the incidence of breast cancer in Africa is lower in comparison to developed countries, mortality from breast cancer is higher [1]. The higher mortality rate has been linked to advanced disease at diagnosis and inadequate treatment [2].

The tumours in African patients show more necrosis, greater nuclear atypia and have higher mitotic activity [3]. Triple-negative breast cancer (TNBC) and human epidermal growth factor receptor 2 (HER2)-enriched breast cancer are more common in African populations and are associated with more aggressive diseases [4].

Metastatic breast cancer (MBC) is a disease that has advanced further than the breast and the regional lymph nodes. MBC can be diagnosed at first presentation (*de novo* disease) or present in patients initially diagnosed with non-metastatic disease (relapsed or recurrent disease) [5]. Recent studies in Kenya have found that 25% of patients diagnosed with breast cancer are diagnosed with metastatic disease at initial diagnosis and more than 50% of patients go on to develop MBC [6].

Several clinical and biochemical factors which affect the outcomes in patients with MBC have been identified. These factors include age, performance status, metastatic site and number of lesions, hazard ratios (HR) status, HER2 receptor status, disease-free interval (DFI) and prior chemotherapy [7].

Five years of survival for MBC in the West ranges from 16% to 28% [8, 9]. There is little data on survival outcomes of breast cancer in Africa and even less on patients with MBC. Studies have reported widely variable 5-year survival rates of MBC of between 8% and 39% [10–13]. These studies were limited by high rates of loss of follow-up and inadequate treatment of metastatic disease.

Due to the higher occurrence, advanced disease and unique tumour biology in the African population, it is important to know the survival outcomes of patients with MBC in our setting [2, 3].

We have a cohort of patients who are on regular follow-up at the breast service and receive chemotherapy and radiotherapy as needed. This addresses the limitations of inadequate treatment and loss to follow-up in most studies from Sub-Saharan Africa.

The purpose of this study was to establish 3 and 5-year survival outcomes of patients with MBC, the clinical and pathological characteristics affecting survival and to describe the treatment modalities used.

## Materials and methods

### Patients

This was a retrospective study conducted at Aga Khan University Hospital Nairobi (AKUHN). This is a private tertiary hospital in Nairobi, Kenya. Most of the patient population are middle and upper-income earners with a tertiary education and majority pay through private insurance. Patients have access to multi-disciplinary care with chemotherapy, radiotherapy and surgical services.

Patients diagnosed and treated for MBC at AKUHN from 1st Jan 2009 to 31st Dec 2017 with complete records were included in the study. Patients with incomplete records or a previous history of another cancer were excluded.

### Data collection

Patient records were reviewed retrospectively.

Survival data collected was:

- Metastasis free survival (MFS): time between first diagnosis of breast cancer and diagnosis of distant metastasis in patients presenting with non-metastatic disease
- Survival (S): time between diagnosis of first metastasis and death
- Overall survival (OS): time between first diagnosis of breast cancer and death

Data on patients’ demographic and clinical characteristics was collected including age, menopausal status, stage at diagnosis, molecular subtype, metastatic site and treatment given.

### Data analysis

The demographic and pathologic characteristics were presented using summary statistics. Continuous variables were shown as medians and ranges and categorical variables shown as percentages and frequencies. Differences between categorical variables and continuous variables were analysed using chi-squared test or Fishers Exact test for categorical data and Student’s *t*-test or Mann–Whitney *U* test for continuous data.

Survival was estimated using the Kaplan–Meier method and compared between the different subgroups of patients using log-rank test. The survival and OS rates were presented as percentages. Median survival time was also calculated.

HRs and 95% confidence intervals (CIs) were calculated by univariable Cox proportional hazard models to assess the relative contribution of age, menopausal status, tumour grade, molecular subtype, metastatic site and treatment type to survival after diagnosis of MBC.

The statistical analysis was carried out using SPSS software (SPSS Inc).

## Results

During the period from 2009 to 2017, 1,210 patients were diagnosed with breast cancer. Out of these, 146 patients were diagnosed with MBC. Among those with MBC, 15 patients were excluded due to incomplete records. A total of 131 patients were included.

### Patient characteristics

The baseline patient characteristics are summarised in Table 1. The median age at diagnosis of breast cancer was 47.00 years (IQR 18). Overall, 30.5% (40) of patients were diagnosed with de novo MBC while 69.5% (91) had progression of disease from an earlier stage.

The most common single metastatic site was bone 58% (76) followed by lung 56.6% (74). Overall, 58.8% (77) of the patients had metastasis to multiple sites with lung plus bone metastasis being the most common sites 11.5% (15) (Table 1).

### Treatment

Out of the 131 patients included in the study, 87.0% (114) received treatment for metastatic disease. Out of these, 63.3% (83) patients had chemotherapy, 57.3% (75) had hormonal therapy and 47.7% (56) had radiotherapy. In those who received treatment for metastatic disease, 53.4% (70) had both chemotherapy and hormonal therapy. Only three of the patients had surgery for metastatic disease; two patients had a ‘toilette’ mastectomy done and one patient had intramedullary nailing for an impending fracture femur (Table 2).

Out of the 91 patients with recurrent disease, 61.5% (56) received systemic chemotherapy for metastatic disease. Among these patients, 15.3% (14) presented with a visceral crisis. Only 46.2% (42) had received prior chemotherapy. In the group receiving chemotherapy for recurrence, 67.9% (38) received taxane-based therapy for metastatic disease while 32.1% (18) received non taxane-based therapy. Capcetabine was the most common single agent used (*n* = 16), (Table 3).

There were seven patients with HER2 enriched disease among the patients with recurrent disease. Of these, only one received herceptin in the adjuvant setting and two patients received herceptin for metastatic disease, (Table 3).

### Survival

**Figure 1.**
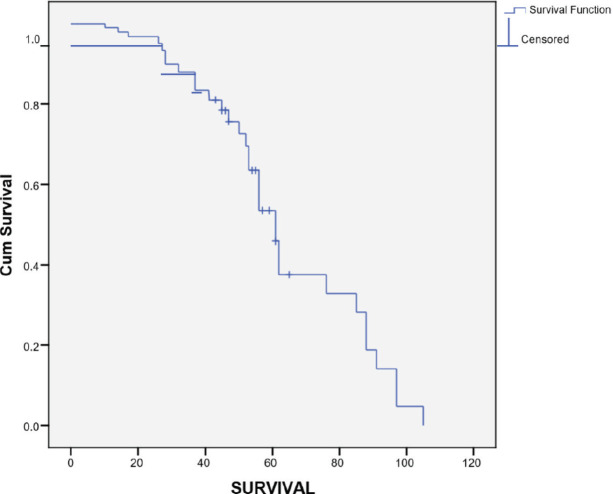
Survival from time from diagnosis of MBC.

The median follow up period was 35 months (range 1–133 months). The survival rate was 31.3% at 3 years and 10.7% at 5 years (Figure 1). The median survival was 22 months (95% CI 14.98–29.01). The OS rate from primary diagnosis of breast cancer was 49.6% at 3 years and 24.4% at 5 years. The median MFS was 17 months (Range 2–105 months).

**Figure 2.**
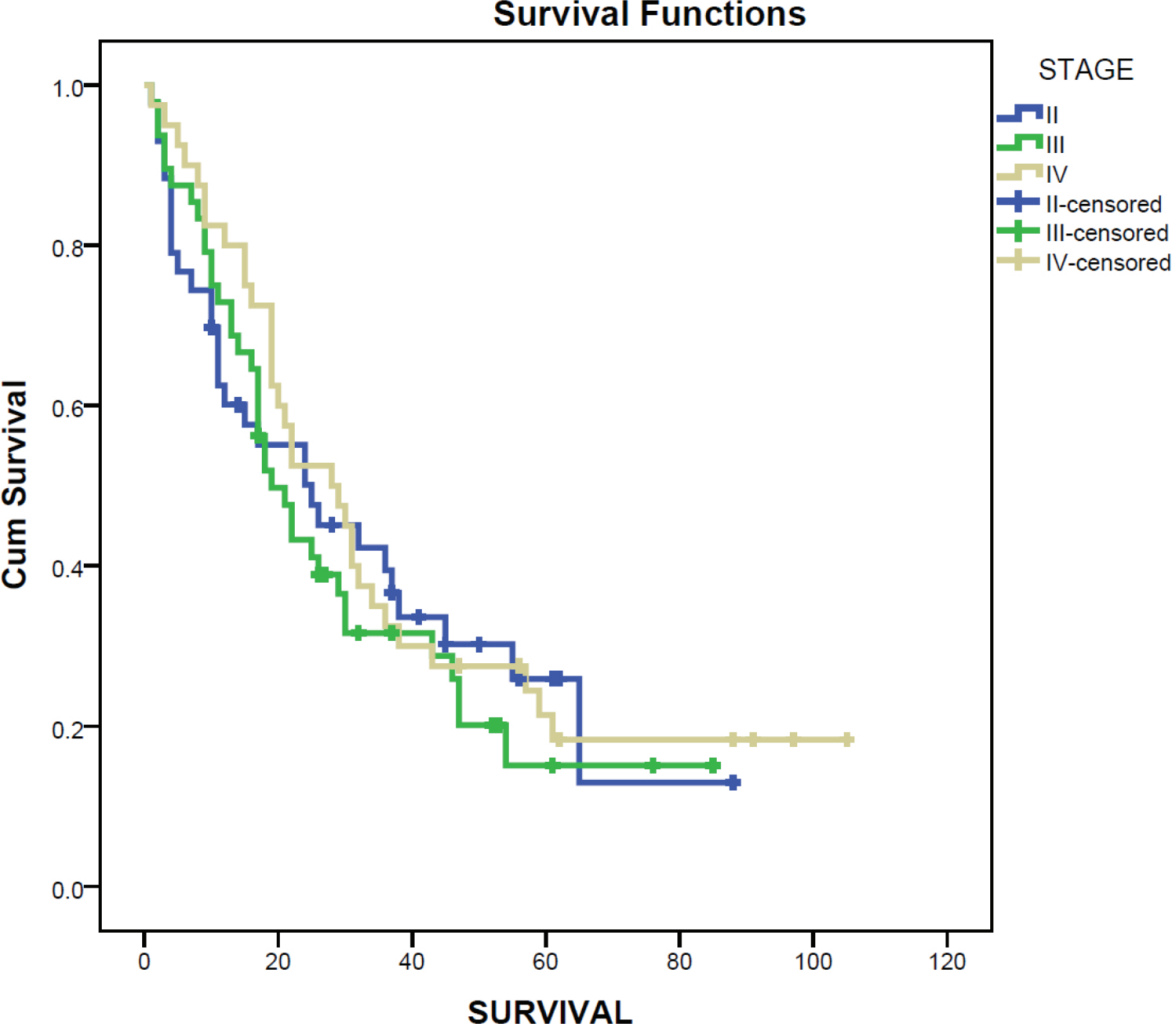
Survival by stage at diagnosis.

Survival after diagnosis of MBC was compared for different molecular subtypes. Luminal A molecular subtype was associated with the longest median survival time at 30 months (95% CI 20.8–39.2) while HER2 enriched disease had the shortest survival at 15 months (95% CI 3.3–26.7). Survival for MBC was also compared for the stage at initial diagnosis using the Kaplan–Meier curve. Those diagnosed at stage IV had the longest median survival at 28 months (95% CI 16.8–39.2) while those initially diagnosed at stage III had the poorest median survival at 19 months (95% CI 13.5–24.5) (Figure 2).

Using the Cox regression model, several potential prognostic factors were evaluated (Table 4). Disease factors which showed a significant positive effect on survival time were Luminal A molecular subtype (HR 0.652 CI 0.473–0.899). Metastasis to the liver or brain was significant negative prognostic factors (HR 0.615 CI 0.413–0.915 and HR 0.566 CI 0.330–0.973, respectively). MFS, age at diagnosis, menopausal status, tumour grade and type of chemotherapy given did not show any statistically significant effect on survival after the diagnosis of MBC on univariate analysis.

## Discussion

Although breast cancer outcomes have improved over the last few decades, MBC is still the predominant cause of breast cancer-specific mortality [14]. Our results showed median survival after diagnosis of MBC of 22 months and 3 and 5-year survival of 31.3% and 10.7%, respectively. This is higher than that reported by Adisa *et al* [12] in Nigeria where they found a survival rate of 27.1% for patients with MBC at 1 year and 8% at 2 years. However, our findings are lower than studies from Uganda which found a 5-year survival rate of 39% for patients with advanced breast cancer (Stage III and IV) between 1996 and 2000 and 37.89% between 2004 and 2012 [10, 11]. Both studies from Uganda had a very large proportion of censored patients due to poor follow up and could have overestimated the cumulative survival. In our study, we included patients who were on regular follow up at our institution and therefore minimizing the bias due to loss of follow up. Similar results to ours were found in a study at a tertiary institution in Tunisia with an OS of 63% at 1 year and 12% at 5 years between the year 2000 and 2007 [13]. In that study, 94.6% of the patients received either chemotherapy, hormonal therapy or both treatments for metastatic disease. In our study, 87% of the patients received treatment for metastatic disease. Our findings are lower than 5-year survival rates for MBC in the West which range from 16% to 28% [8, 9]. This may be due to limitations in access to treatment such as anti-HER2 therapy due to high cost. Higher rates of TNBC and high grade disease may also account for the poorer outcome observed in our study. Our findings are comparable to those of African-American women in a surveillance, epidemiology and end results (SEER) database analysis from 2001 to 2011 which found a median survival of 22 months and a 5-year survival of 11% for African-American women with MBC [15]. This may be explained by similar tumour biology in our patients and African-American women.

The median age at diagnosis of BC was 47 years. This is more than a decade lower than that of Western populations where the median age at diagnosis is 62 years [16]. Advanced age at diagnosis of MBC is associated with poor outcomes. Population-based studies in Europe and America have found that patients over 70 years had higher mortality than those 50–69 years and below 50 years [5, 17–19]. In our analysis, we did not find age at diagnosis to be a significant prognostic factor. This may be due to a small sample size.

In our study, 58% (76) of our patients had bone metastasis. Patients with bone metastasis have up to twice longer survival than those with visceral metastasis [20–22]. Our study did not find bone metastasis to affect prognosis (HR 1.212 CI 0.815–1.803 *p* = 0.343). However, metastasis to the liver and brain was a significant negative prognostic factor (HR 0.615 CI 0.413–0.915 and HR 0.566 CI 0.330–0.973, respectively). This is in keeping with a SEER database analysis of 7,575 patients between 2010 and 2013 which found liver and brain metastasis to be a negative prognostic factor whereas lung and bone metastasis had no significant effect on survival [23]. Patients with bone metastasis may benefit from treatments such as radiotherapy and bisphosphonates whereas there is no specific therapy for brain and liver metastasis. This may account for the differences in outcomes.

Hormone receptor status is a significant prognostic factor in MBC [24]. In our study, we only found the Luminal A molecular subtype to be positively associated with survival (HR 0.652, 95% CI 0.473–0.899). Estrogen receptor (ER)/progesterone receptor (ER) positive tumours have been shown to have lower proliferation and thus lower grade [25]. They are amenable to hormonal therapy and this may explain the better survival outcomes in these patients [26]. Even though HR-positive and HER2-positive disease has been associated with a more favourable outcome, our study found the lowest median survival in patients with Luminal B and HER2 enriched molecular subtypes (median survival 17 and 15 months, respectively). This may be explained by inadequate treatment with trastuzumab. Out of 25 patients with either Luminal B or HER2 enriched molecular subtypes, only eight patients received trastuzumab therapy. This was due to the high cost of trastuzumab.

MFS has been found to be a prognostic factor in MBC. In a study examining the survival of 3,524 patients with MBC, Dawood *et al* [5] found that those with MBC at initial diagnosis had better survival outcomes than those with relapsed disease (27.2 versus 39.2 months, *p* < 0.0001). However, patients with relapse who had a DFI of more than 5 years had better survival than those with MBC at initial diagnosis [5]. In our patients, those with MBC at initial diagnosis had a better survival of 28 months (95% CI 16.8–39.2) than those initially diagnosed with stage II or III disease (25 months, 95% CI 7.2–42.8 and 19 months, 95% CI 13.5–24.5, respectively). The theory of clonal evolution due to adjuvant chemotherapy in those with recurrent disease may explain these findings. Studies have suggested that the use of chemotherapy eliminates sensitive clones and recurrent disease consists of resistant cells [27]. In a study of 815 patients with MBC, Lobbezoo *et al* [28] found no significant difference in survival between patients with MBC at initial diagnosis and those with relapse with a DFI >24 months. However, those with DFI <24 months had a poorer survival than those with de novo MBC. Patients with a longer progression free survival are likely to have less aggressive tumours and therefore a better prognosis [29].

With earlier detection of breast cancer and use of adjuvant chemotherapy, most patients with MBC will have been exposed to prior systemic chemotherapy. Taxanes have been shown to be effective in patients previously treated with anthracyclin-based chemotherapy [30]. A systematic review of taxane-based therapy for MBC found that taxane-based therapy improved OS in patients with MBC compared to non-taxane-based therapy [31]. However, there was significant heterogeneity among the trials due to the varying comparator regimens. Although we found a better survival in patients who received taxane-based therapy compared to non-taxane based therapy, the difference was not statistically significant. This may be due to a small sample size.

## Limitations

The limitations of our study were small sample size and the retrospective study design. Some records such as tumour grade and chemotherapy regimen in some of the charts were missing. During the period of this study, cyclin-dependent kinase 4/6 inhibitor therapy was not standard of care and was not given to the patients. Molecular tests such as phosphoinositide 3-kinase mutations, breast cancer susceptibility gene 1 or 2 mutations (BRCA1 OR BRCA2) and PD-L1 expression were not done and the relevant targeted therapy was not given.

## Conclusion

Our study found survival rates for patients diagnosed with MBC to be lower compared to studies from Western countries but higher than studies from Sub-Saharan Africa. Survival rates were similar to those of African American women. Luminal A molecular subtype was a significant positive prognostic factor and metastasis to the liver or brain were found to be significant negative prognostic factors. Improved access to adequate treatment for MBC is required in the region.

## Recommendation statement

Further studies are needed to look into the relationship between the diagnoses of de novo versus relapsed MBC and its impact on the survival outcome. Larger prospective studies on associations of risk factors and breast cancer subtypes with survival outcomes are also recommended.

## Ethics statement

The Aga Khan University Research Ethics Committee gave ethical clearance (Reference number 2020/IERC-46 (v2)).

Patients or the public were not involved in the design, conduct, reporting, or dissemination plans of our research.

## Contributorship statement

All authors contributed to the design and implementation of the research, to the analysis of the results and to the writing of the manuscript.

## Conflicts of interest

There are no competing interests for any author.

## Funding

There is no funding to report for this submission.

## Figures and Tables

**Table 1.** Patient characteristics.

Characteristics	All (*n* = 131)	*N*
Median age at diagnosis of breast cancer	47.00 years	131
Menopausal status		
Post-menopausal	42%	55
Pre-menopausal	58%	76
Stage at diagnosis		
II	32.8%	43
III	36.6%	48
IV	30.5%	40
Molecular subtype		
Luminal A	58%	76
Luminal B	12.2%	16
HER2 enriched	6.9%	9
Triple negative	19.8%	26
Unknown	3.1%	4
Histological type		
IDC	93.1%	122
ILC	6.9%	9
Tumour grade		
I	3.1%	4
II	47.3%	62
III	48.1%	63
Missing	1.5%	2
Metastatic site		
Single site Lung Bone Liver Brain Other	41.2% 11.5% 16.0% 9.9% 2.3% 1.5%	54 15 21 13 3 2
Multiple sites Lung and bone Lung and liver Liver, lung and bone Liver and bone Brain and bone Lung and brain Bone, brain and liver Brain, lung and bone Brain, lung and liver Brain and liver Other	58.8% 11.5% 10.7% 9.9% 6.9% 2.3% 2.3% 2.3% 1.5% 0.8% 0% 10.7%	77 15 14 13 9 3 3 3 2 1 0 14

**Table 2.** Treatment of metastatic disease.

Treatment modality	Percentage (*N* = 131)	*N*
Surgery	2.3%	3
Chemotherapy Taxane Non-taxane	63.4%	83 37 46
Radiotherapy	42.7%	56
Hormonal therapy	57.3%	75
Trastuzumab	6.1%	8

**Table 3.** Treatment of recurrent disease.

Variables		*N*	Percentage
Recurrent disease		91	100
HR positive		63	69.2
Visceral crisis		14	15.3
Hormonal therapy		46	50.5
Adjuvant	Metastatic		
Tamoxifen	AI	2	
AI	Tamoxifen	2	
Tamoxifen	Tamoxifen	25	
AI	AI	7	
None	Tamoxifen Or AI	10	
Tamoxifen Or AI	None	12	
Systemic chemotherapy		56	61.5
Adjuvant	Metastatic		
Taxane	Taxane	22	
Taxane	Non taxane	9	
Non taxane	Taxane	16	
Non taxane	Non taxane	9	
Monotherapy		34	
Capecitabine		18	
Taxane		12	
Gemicitabine		4	
Combination therapy		22	
Her2 enriched		7	7.7
Adjuvant herceptin		1	
Metastatic herceptin		2	

**Table 4.** Prognostic factors.

	HR	95.0% CI		*p* value
		Lower	Upper	
MFS	0.995	0.984	1.006	0.353
Age	0.989	0.973	1.005	0.184
Menopausal status				
Pre-menopausal	1	-	-	-
Post-menopausal	0.888	0.724	1.088	0.252
Tumour grade				
Grade 1	1.158	0.593	2.261	0.667
Grade 2	1.008	0.681	1.492	0.968
Grade 3	1	-	-	-
Molecular subtype				
HER2	1.471	0.845	2.561	0.172
Luminal A	0.652	0.473	0.899	0.009
Luminal B	1.219	0.767	1.938	0.403
TNBC	1	-	-	-
Metastatic site				
Lung	0.935	0.626	1.397	0.744
Liver	0.615	0.413	0.915	0.017
Brain	0.566	0.330	0.973	0.040
Bone	1.212	0.815	1.803	0.343
Other	1.231	0.620	2.446	0.553
Single site	1.226	0.999	1.504	0.051
Multi sites	1	-	-	-
Chemotherapy				
Non taxane	1.180	0.740	1.879	0.487
Taxane	0.858	0.520	1.415	0.549
No chemo	1	-	-	-
